# Transmembrane Helix Dynamics of Bacterial Chemoreceptors Supports a Piston Model of Signalling

**DOI:** 10.1371/journal.pcbi.1002204

**Published:** 2011-10-20

**Authors:** Benjamin A. Hall, Judith P. Armitage, Mark S. P. Sansom

**Affiliations:** Oxford Centre for Integrative Systems Biology, Department of Biochemistry, University of Oxford, Oxford, United Kingdom; University of Houston, United States of America

## Abstract

Transmembrane α-helices play a key role in many receptors, transmitting a signal from one side to the other of the lipid bilayer membrane. Bacterial chemoreceptors are one of the best studied such systems, with a wealth of biophysical and mutational data indicating a key role for the TM2 helix in signalling. In particular, aromatic (Trp and Tyr) and basic (Arg) residues help to lock α-helices into a membrane. Mutants in TM2 of *E. coli* Tar and related chemoreceptors involving these residues implicate changes in helix location and/or orientation in signalling. We have investigated the detailed *structural* basis of this via high throughput coarse-grained molecular dynamics (CG-MD) of Tar TM2 and its mutants in lipid bilayers. We focus on the position (shift) and orientation (tilt, rotation) of TM2 relative to the bilayer and how these are perturbed in mutants relative to the wildtype. The simulations reveal a clear correlation between small (ca. 1.5 Å) shift in position of TM2 along the bilayer normal and downstream changes in signalling activity. Weaker correlations are seen with helix tilt, and little/none between signalling and helix twist. This analysis of relatively subtle changes was only possible because the high throughput simulation method allowed us to run large (n = 100) ensembles for substantial numbers of different helix sequences, amounting to ca. 2000 simulations in total. Overall, this analysis supports a swinging-piston model of transmembrane signalling by Tar and related chemoreceptors.

## Author Summary

Understanding the physical mechanisms by which signals are transduced across a lipid bilayer is central to an account of cell signalling. We have used a novel technique for performing simulations to model the interactions of transmembrane (TM) helices from bacterial methyl accepting chemoreceptor proteins with lipid bilayer membranes. These chemoreceptor signalling proteins dictate bacterial behaviour by biasing the movement of the cell towards food sources and away from toxins. The mechanism of signalling by these proteins through the membrane remains unclear, despite structural characterization of different components. Researchers have generated a large number of mutants in the TM2 helix, but the precise changes in helical behaviour which are induced by these mutants are not always clear. Our simulation approach allows the consequences of mutations to be unambiguously interpreted in terms of changes of position and orientation of the TM2 helix. Our results for the receptors Tar and Trg support a “swinging piston” model of signalling where changes in tilt and position of the helix relative the bilayer confer signals. This approach may be extended to a range of TM signalling events different classes of receptors and thus represents an advance in the interpretation of TM helix mutagenesis experiments.

## Introduction

Signalling by transmembrane (TM) receptor proteins is central to the biology of both bacterial and eukaryotic cells. For many receptors (other than the GPCRs) the TM domain is relatively simple, consisting of just one or two TM helices per protein subunit. In these receptors changes in TM helix position and orientation relative to the membrane lipid bilayer almost certainly play a key role in the mechanism of signalling across the membrane (for a recent review see [1]). However, because of the difficulties of determining high resolution structures of intact receptor proteins, our understanding of such mechanisms is incomplete. By combining functional data with computational biophysical descriptions of receptor TM helix interactions with membranes we can better understand these complex membrane systems.

**Figure 1:**
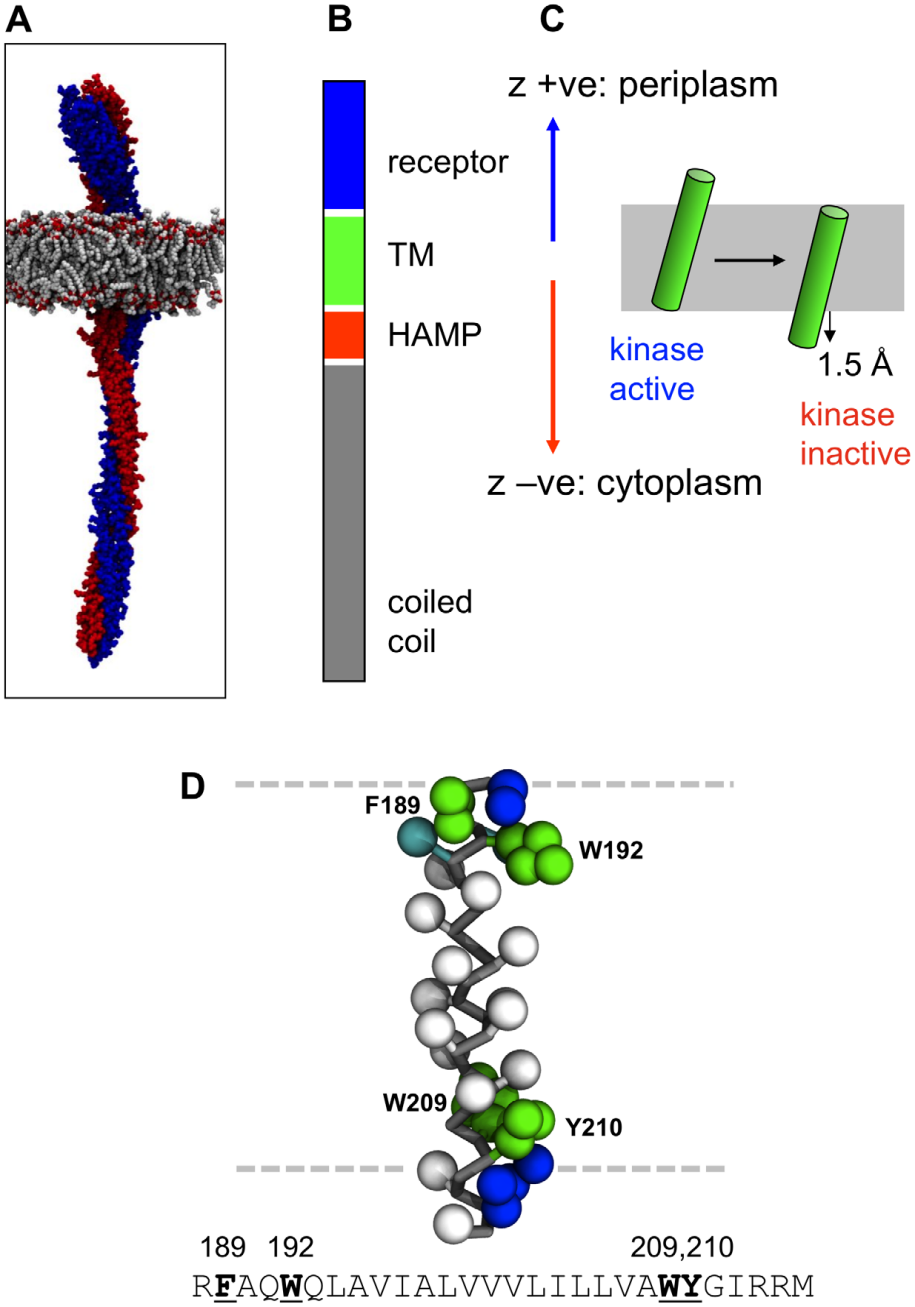
The Tar chemoreceptor and its TM2 helix. **A** Structural model of a chemoreceptor (Tsr) dimer (the two subunits in red and blue) in a phospholipid bilayer (grey). **B** Schematic of the domains of the Tar receptor, showing the periplasmic receptor (blue), transmembrane (TM; green), and cytoplasmic HAMP (red) and coiled-coil (grey) domains. **C** Illustration of the piston model of signalling via displacement of the TM helix (green) relative to the lipid bilayer (grey). The z-axis convention used in subsequent figures is defined, with a positive displacement corresponding to movement in the periplasmic direction and a negative displacement to movement towards the cytoplasm. **D** The Tar TM2 helix structure in coarse-grained representation. Apolar residue sidechains are represented as white spheres, polar sidechains as cyan spheres, aromatic sidechains as green spheres, and basic sidechains as blue spheres. The peptide backbone is drawn as grey bonds. The approximate location of the lipid bilayer is indicated in by the broken horizontal lines.

Bacterial chemotaxis receptors (chemoreceptors) provide a well-studied example of such systems. Structural studies of *E. coli* chemoreceptors [2], [3], [4], [5], [6] have shown that e.g. the Tar receptor consists of an extracellular (periplasmic) receptor domain and an α-helical TM domain. The latter is linked via a HAMP domain to a long intracellular coiled-coil domain (Fig. 1AB) (HAMP is a linker domain present in Histidine kinases, Adenyl cyclases, Methyl-accepting proteins and Phosphatases which is found in bacterial sensor and chemotaxis proteins and in eukaryotic histidine kinases.) The receptor is dimeric, and the homodimers seem to be arranged in higher order structures based on a hetero-trimer of homodimers unit [6]. Signalling occurs via ligand binding to the extracellular receptor leading to a change in interactions of the intracellular coiled-coil domain with CheW (an adaptor protein) and CheA (a histidine kinase). For example, in Tar the binding of a chemoattractant (aspartate) to the receptor is thought to causes a conformational change which is propagated through the transmembrane (TM) domain to the signal processing HAMP domain. This in turn cause further changes which propagate from individual dimers, altering the packing and orientation in trimer of dimers structures (as observed by cryoelectron microscopy tomography, [6]), and from there to a wider, mostly hexagonal array consisting of MCPs, histidine kinase CheAs and adaptor CheW proteins [7]. This “conformational spread” of information from individual receptors through an array creates a highly sensitive amplification system with a high signal to noise ratio [8], [9].

The nature of the conformation changes in the receptor in response to ligand binding events are not clearly understood, despite a wealth of structural and genetic information. Attractant binding at the receptor domain causes motions in the second transmembrane helix, TM2, which links the receptor domain to the HAMP domain. The coiled coil region is able to undergo other internal dynamic changes on activation of the receptor. Together, these conformational changes cause a reduction in the activity of the histidine kinase CheA, which alters downstream signalling events [10], [11].

The dynamic behaviour of the TM2 helix in signalling is the subject of on-going debate. Studies of aspartate chemoreceptor Tar suggest that on binding of attractant to the receptor domain TM2 undergoes a 1.5 Å piston-like motion in the membrane, towards the cytoplasm. This was initially proposed from lock-on/lock-off disulphide mutants [12] and identified from the crystal structures of the Tar apo and ligand bound forms of the periplasmic domain [13]. It was subsequently supported by solid state NMR measurements, spin labelling measurements, and data from mutagenesis studies [14], [15], [16]. However, a rotational mechanism has also been invoked on the basis of studies of the HAMP domain [4], [6]. In order to dissect out the elements of the transbilayer signalling mechanism of Tar and related chemoreceptors, it is important to establish how TM2 interacts with a lipid bilayer membrane, and how mutations in TM2, which perturb signalling, alter such interactions. This behaviour has also been studied extensively in the minor chemoreceptor Trg, using genetic and simulation techniques [17], [18], [19], [20], revealing the conservation of the piston model of signalling across related proteins.

Central to a piston-like model (Fig. 1C) is the question of how the TM2 helix is positioned/oriented relative to a lipid bilayer and how changes in this position/orientation may be used to signal across a membrane. This is related to the more general question of what factors ‘lock’ a TM helix into position in a bilayer. This has been extensively studied using synthetic model TM helices [21], [22], [23], [24], [25], with an emphasis on the roles of tryptophan (W) and arginine (R) sidechains, mirroring their importance in the structures of more complex membrane proteins and their interactions with lipids [26], [27], [28], [29]. Alongside experimental approaches, molecular dynamics simulations of these helices have provided insights into folding processes in membranes and the precise interactions made between individual residues and the membrane [25], [30], [31], [32], [33], [34]. Typically such simulations employ full atomistic detail, which is CPU intensive and therefore limits the statistical significance of the observed behaviour. In contrast, coarse grain (CG) simulations by reducing the level of detail in a system (by a factor of roughly 4∶1 in the case of MARTINI [35], [36] and related CG models [37], [38], [39]) yield an effective increase in computational speed of between 1 and 2 orders of magnitude. A number of studies on synthetic peptides have shown the CG-MD can accurately reproduce the experimentally observed positions and orientations of such helices in lipid bilayers [25], [31], [32], [38], [40], [41]. To facilitate the running and analysis of large and multiple ensembles of CG-MD simulations, we have recently developed a high throughput approach [25], [42]. With this approach, we have shown that the improved sampling of helix/bilayer interactions made possible by such ensembles of simulations allows, for example, the helix/bilayer interactions of synthetic model peptides as seen in solid state NMR to be probed [25], [42].

Here we use comparative CG-MD simulations enabled by our high throughput simulation pipeline to understand the structural consequences of several sets of mutations ([20], [43], [44], [45], supplementary table S1) in the TM2 of two chemoreceptors Tar, the high abundance aspartate receptor from *E.coli* and Salmonella, and Trg, the low abundance ribose chemoreceptor from *E.coli*. We build simulations including a roughly 30 residue helix representing TM2 in an explicit membrane with explicit solvent. The outcome of this analysis supports a piston-like model of TM signalling, whilst also highlighting a potential role for helix tilt in Tar.

## Results

### Tar TM2 C-terminal aromatic scanning mutants

**Figure 2:**
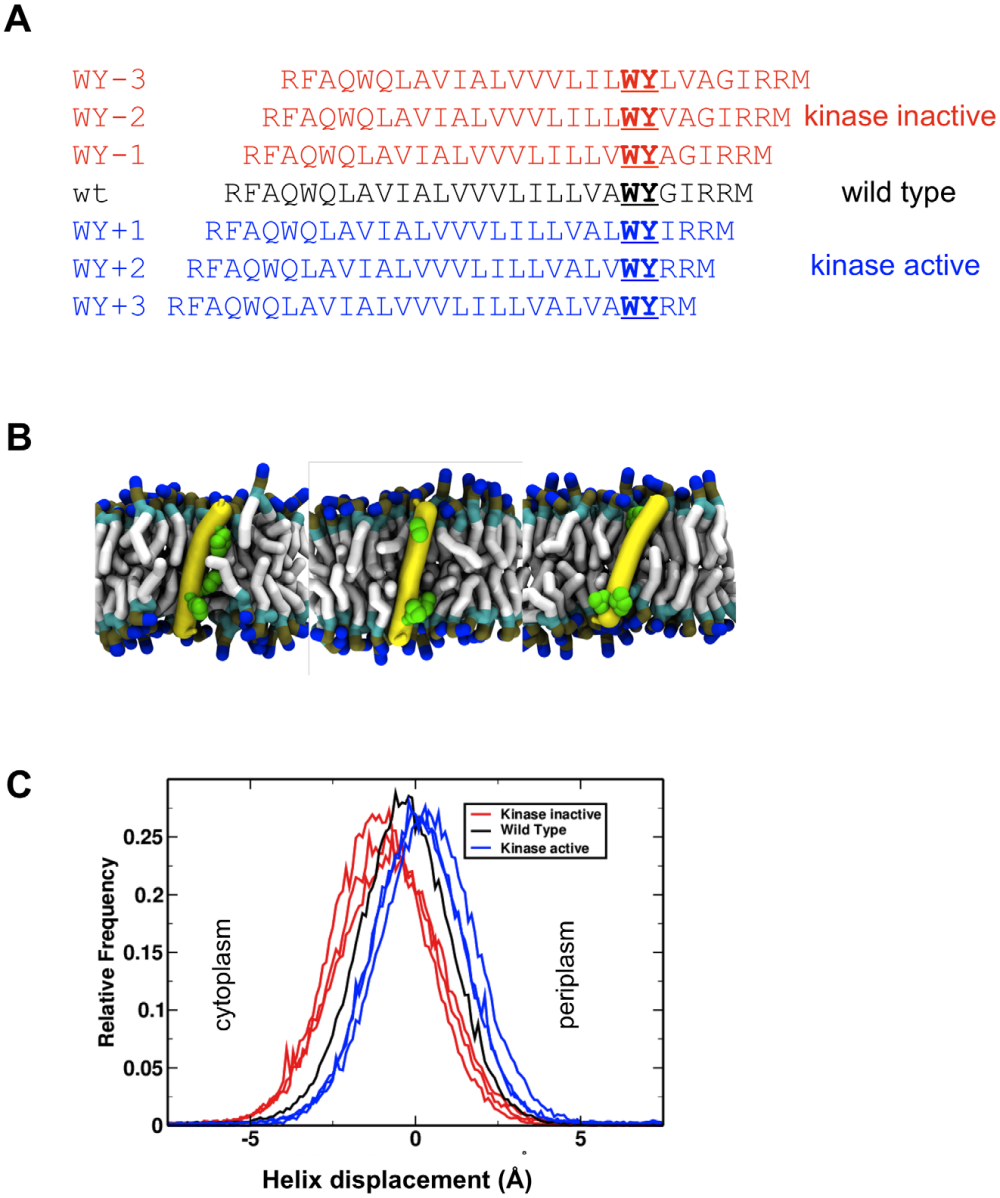
Tar TM2 C-terminal aromatic scanning mutants. **A** Sequences of WY-scanning mutants of the *E. coli* Tar TM2 helix are shown aligned to maintain the position of the C-terminal aromatic motif (WY; underlined). Three mutants with an inactive kinase (determined from flagellar rotation behaviour [44]) which are anticipated to have the TM2 helix translated towards the cytoplasm direction are in red, the wild type sequence in black, and three mutants which are anticipated to translate the TM2 helix in a periplasmic direction are in blue. **B** Tar TM2 WY scanning mutants, show helix reorientation in the membrane. Snapshots of WY-3, wild type and WY+3 helices (left to right), demonstrating interactions of aromatic residues (in green) to the interfacial region. The phospholipid hydrophobic tails are in pale grey, and their phosphate groups in brown. The peptide helix is shown as a yellow tube with the aromatic sidechains in green. **C** Helix displacements with respect to the bilayer centre of the mutant TM2 helices. Kinase inactive mutants (red) are displaced towards the cytoplasm, and kinase active mutants (blue) are displaced towards the periplasm. Histograms shown are for all time points of all members of each ensemble (excluding rare interfacial events).

Aromatic sidechains (specifically tryptophan, W, and tyrosine, Y) have been shown to play a key role in anchoring TM helices in a given position in membranes, through H-bonding interactions to the phospholipid carbonyl oxygens in the interfacial region of the bilayer [23], [46]). Thus, by mutating/inserting aromatic residues it should be possible to modulate/tune the location/behaviour of a TM helix in a membrane [30], [47]. This approach was applied in a set of studies [43], [44] in which the signalling behaviour of Tar from *E. coli* was modulated by performing an asymmetric scan of the cytoplasmic anchoring WY sidechain pair (W209/Y210; see Fig. 2A) in the TM2 helix. By shifting the WY pair up to 3 residues towards either the C-terminal or N-terminal end of the TM2 helix (WY+3 to WY−3 respectively in Fig. 2A), it was shown that downstream signalling and flagellar behaviour correlated with the position of the aromatic anchors.

We mimicked these WY-scan experiments by performing simulations in which the TM2 helix was altered to correspond to the WY-scan mutants. Self-assembly simulations were then performed (ca. 100 per TM2 sequence) and the resultant helix/bilayer systems (Fig. 2B) analysed in terms of helix position and orientation relative to the phospholipid bilayer.

Analysis of the WY scans (Fig. 2C), reveals that the mutations induced changes in position, tilt and rotation of the transmembrane helix (Fig. 2C, supplementary Fig. S1) relative to the lipid bilayer. Visualisation of the simulation trajectories suggests that the interactions of the WY pair with the lipid headgroups dictates the position and dynamics of the helix in the membrane. Both the wild type and mutants helices adopted a transmembrane orientation, as expected, and so statistical analysis of the simulation ensembles was required to dissect out the relatively subtle impacts of the WY-scan mutations on helix bilayer interactions. From such analysis (Fig. 2C) it was evident that the signalling activity of the mutants correlates quite clearly with changes in the position of the mutant helices relative to the wild type ensemble. In particular reduced kinase activity mutants result in a *cytoplasmic* shift of the TM2 helix in the bilayer relative to the wild type, whereas increased kinase activity mutants resulted in a *periplasmic* shift.

In addition to the correlation between signalling state and helix translation there was some evidence for changes in helix orientation (e.g. two of the reduced kinase activity mutants, WY−2, WY−3 appear to have an increased helix tilt angle relative to the bilayer normal; supplementary Fig. S1A). However no clear patterns emerged relating the activities of the mutants with helix rotation (about the helix axis) suggesting that such rotations may not play a role in signalling (supplementary Fig. S1B). It is noteworthy that the difference in position relative to the bilayer centre of the reduced and increased kinase activity mutant helix populations is ca. 1.5 Å (with a change in helix tilt angle of ca. 5°), which matches the crystallographically determined displacement of the connecting helix of Tar TM2 from a number of sources [12], [13], [15], [16].

**Figure 3:**
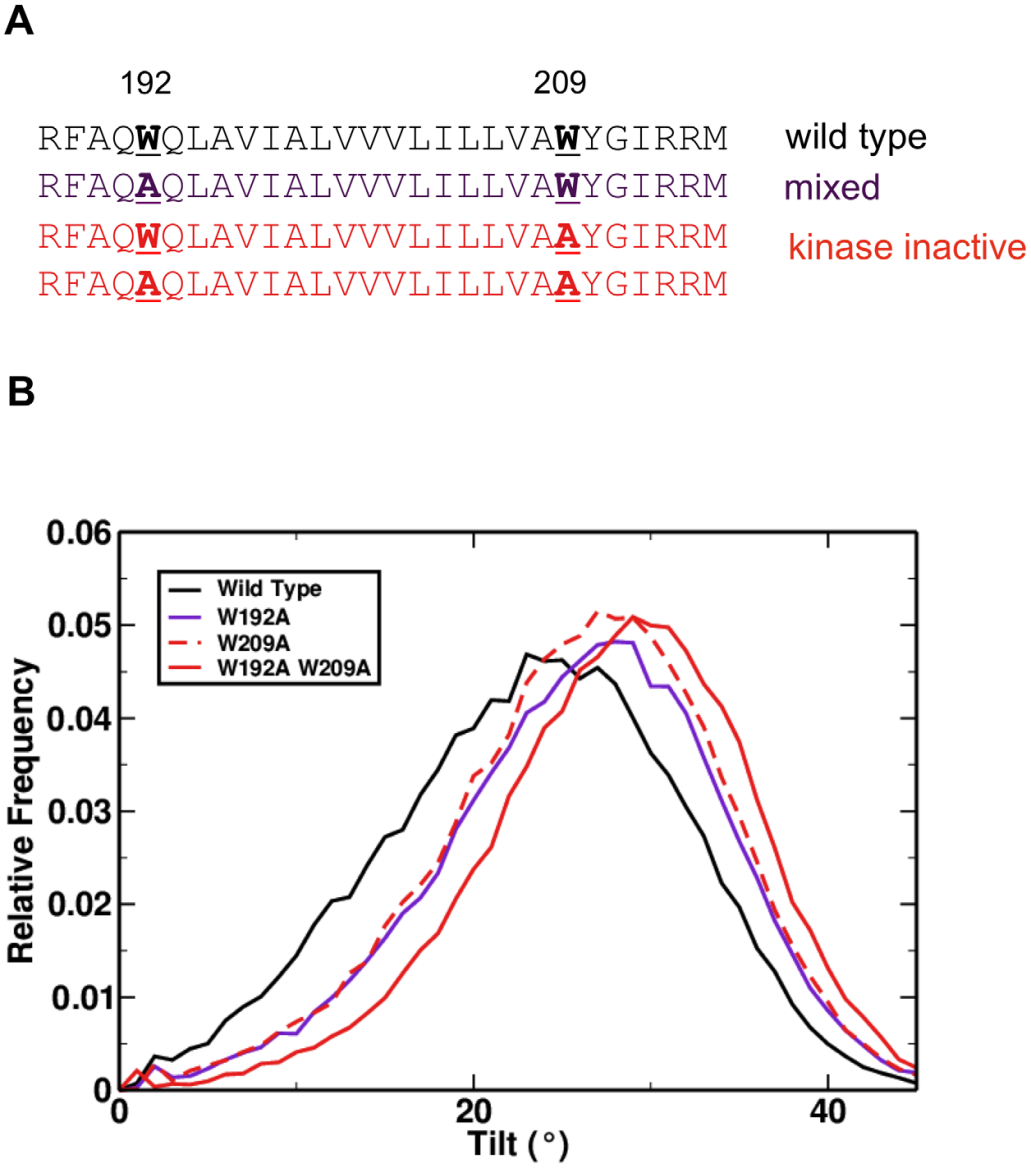
Position and orientation of Tar TM2 tryptophan to alanine mutations [**43**]. **A** Sequences and phenotypes of mutants. **B** Tilt distributions of mutant helices relative to the bilayer normal, revealing an additive effect of mutating individual tryptophan residues to alanine, with individual mutants causing a ∼2.5° increase in tilt and the double mutant a ∼5° increase.

Point mutations of the helix anchoring tryptophan residues (W192 and W209; Fig. 3A) to alanine in TM2 of Tar have also been studied [43]. Visualisation of simulations corresponding to these mutations reveals that the helices also remain transmembrane and do not undergo major changes in orientation. Quantitative analysis of single point mutations (i.e. W192A and W209A) showed in each case a ca. 2.5° increase in the tilt of the helix relative to the membrane, and an increase of ca. 5° for the double mutation (W192A, W209A; Fig. 3). These changes in tilt correlate with changes in signalling activity. In contrast, shifts relative to bilayer of these tryptophan to alanine mutations are minimal (ca. 0.2 Å; supplementary Fig. S2A). This suggests that there may be a role for *both* shift and tilt of helices in the signal transduction process.

### Tar TM2 arginine scanning mutants

Arginine and lysine sidechains may also play important roles in anchoring transmembrane helices, via ‘snorkelling’ interactions with the phosphate moiety of lipid headgroups [24], [25], [47], [48], [49]. This interaction might be anticipated to be somewhat stronger than that of tryptophan and tyrosine. As such, replacement of anchoring aromatic residues with arginine residues would be expected to draw the helix towards one side of the bilayer. Furthermore, introduction of basic residues into the core of the hydrophobic helix may be expected to alter the rotation of the helix due to the strong barrier to placing cationic groups within the bilayer core [25]. Within this context, we simulated helices which correspond to two sets of studies in which basic residue were introduced into TM2. Thus, a site directed mutagenesis approach has been described in which arginine was introduced into the interfacial region of Tar from *Salmonella* [45], and a genetic screen of *E.coli* Trg identified two cases of a basic residue introduced into the bilayer core [20].

**Figure 4:**
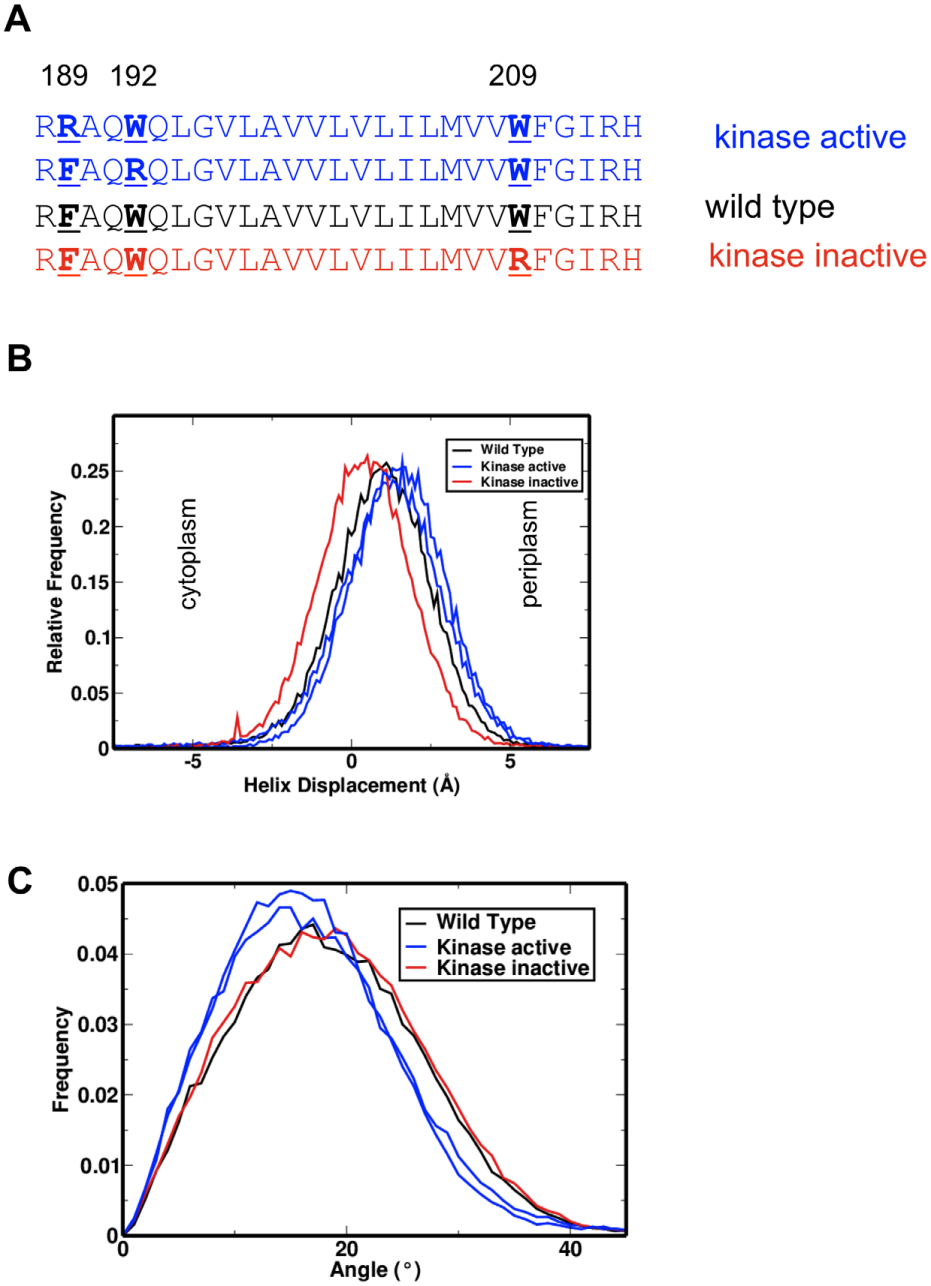
Tar TM2 arginine scanning mutants [**45**]. **A** Sequences and phenotypes of arginine scanning mutants of the *Salmonella typhimurium* Tar TM2 helix are shown with the inserted arginine residue underlined and aligned to maintain the position of the C-terminal aromatic motif (WY; underlined). Two mutants (F189R and W192R; blue) have an active kinase and are anticipated to have the TM2 helix translated towards the periplasm; the inactive kinase mutant (W209R; red) is anticipated to translate the TM2 helix in a cytoplasmic direction. **B** Helix displacements with respect to the bilayer centre of the arginine mutant TM2 helices. The kinase inactive mutant (W209R; red) is displaced towards the cytoplasm, and the kinase active mutants (F189R and W189R; blue) are displaced towards the periplasm. Histograms shown are for all time points of all members of each ensemble (excluding rare interfacial events). **C** Helix tilt distribution relative to the bilayer normal, revealing a reduced tilt in the kinase active mutants (F189R and W189R; blue) and an unchanged tilt in the kinase inactive mutant (W209R; red).

Three key arginine-introducing mutations of Tar from *S. typhimurium* have been studied to exemplify reduced/increased kinase activity phenotypes: F189R and W192R (increased kinase activity) and W209R (reduced kinase activity; see Fig. 4 and supplementary Fig. S3). In the simulations, W209R modified-helices are displaced towards the cytoplasm relative to wild type, and have a minimally altered tilt angle in the membrane. In contrast, W192R and F189R both show slight displacement towards the periplasm, and show a reduced tilt angle, consistent with the observed changes in tryptophan to alanine point mutants (where increased inhibition is associated with increased tilt). Examination of rotation angles shows no clear link between rotation and kinase activity, though W192R has a distinctly reduced angular preference.

A genetic screen of Trg revealed three basic mutations in the bilayer core: T215K and T215R (increased kinase activity) and L216R (reduced kinase activity) [20]. Simulations of the corresponding TM2 helices (Fig. 4) indicate that in all three cases the helix is shifted towards the cytoplasm. Significantly, the reduced kinase activity mutant (L216R) shows an increased tilt in the membrane relative to wild type. Furthermore the two increased kinase activity mutants are rotated (i.e. a change in helix twist) by ca. 180°. This is significantly greater than the 26° rotation proposed to confer signals through the membrane [4]. It is likely that this extreme rotation change, coupled with the strong angular dependence in this specific pair of mutations, is leading to an increased kinase activity phenotype through reorienting the signalling helix to a state which is not accessible in the wild type. Additionally, this could also reflect novel interactions created within the transmembrane helix bundle, which cannot be explored in this single helix system.

## Discussion

In this study we have shown that a high throughput approach to CG-MD simulation of TM helices and their interactions with lipid bilayers allows the integration and interpretation of a large body of mutational data on bacterial chemoreceptors. Using simulations to examine a wide variety of signalling mutations from the past two decades of chemotaxis research [20], [43], [44], [45] we have found that the majority of the data are consistent with a swinging piston mechanism for signal transduction by Tar-like chemoreceptors. In particular, our results suggest that signalling through the membrane is dominated by translational motions of helix TM2, with some additional contribution from changes in helix tilt. This has been most clearly demonstrated for the WY scanning mutations of Tar from *E. coli* (Fig. 2), but is also supported by data from a range of additional TM2 mutations.

Alongside such mutational data, there exists a substantial body of knowledge on the role of aromatic and basic sidechains in determining the position, orientation and dynamics of synthetic model TM helices in lipid bilayer membranes [23], [50]. Many of the mutations described in this study relate directly to the alteration of the anchoring residues which are directly studied in these approaches, and highlight the importance of understanding interactions of peptides with the membrane through the comparison of TM helices in signalling proteins and synthetic peptides. Here again alongside experimental approaches, simulations of the helices have provided insights into folding processes in membranes and the precise interactions made between individual residues and the membrane [25], [30], [31], [40]. For example, the snorkelling behaviour of arginine in the membrane has been extensively studied in model peptides [24], [25] revealing that the effect of snorkelling by arginine on helix orientation is dependent on location of the arginine in the membrane. Taken together, these results suggest that MD simulations may be used not only to help establish the underlying principles of the interactions of transmembrane helices with their lipid bilayer environment, but also that simulations may be used to probe dynamic shifts in TM helix location and orientation which underlie signalling events in complex receptor systems.

The approach we have described has some limitations. In particular, we neglect possible roles of helix packing interactions within the TM domain and their possible influence on signalling via the TM2 helix. There are some indications in the literature for possible roles of TM1-TM2 and TM2-TM2 interactions. Substitution of a isoleucine with a phenylalanine in *E. coli* Tar TM2 can cause a significant change in receptor activation [51]. Additionally, in the TM2 helices of some chemoreceptors from *R. sphaeroides* there is a (small)XXX(small) helix dimerization sequence motif [52], suggestive of relatively strong interactions between the TM helices of this system. However, mutagenesis of the hydrophobic membrane core of Tar TM2 has revealed a tolerance to a wide range of different amino acid substitutions including large to small, small to large, and non-polar to polar [53]. Future studies will examine the interactions of the helices within the bundle to better define the nature of these interactions, and should include the activity lock mutations from the literature (such as those described in [12] and reviewed in [14]). Furthermore, future studies on larger structures may wish to take into account the restraining effects of the complete receptor on the position and orientation of the helix in the membrane.

Whilst our simulations have indicated that changes in position and, to a lesser extent, orientation of TM2 induced by mutants alter signalling, it remains uncertain how these local helix motions may alter the conformation of the chemoreceptor protein as a whole. In particular, future studies will need to include more complete models of the structure of the entirety of Tar in order to understand e.g. the effect of TM2 helix motions on the adjacent HAMP domain and its interactions [4].

This study highlights the value of simulation of the dynamic structural implications of TM helix mutants to aid interpretation of experimental data on signalling systems. Through the close examination of a wide variety of different datasets it is possible to discern similarities in effect of highly distinct modifications to the wild type signalling helices. Whilst we have developed this approach in the context of bacterial chemosensing, it could be readily adapted to e.g. simulation of helix dimerization by TM helices of mammalian receptor tyrosine kinases [54] for which there is considerable evidence for the role of TM helix mutations in disease [55].

## Methods

### CG-MD simulations

Coarse grained molecular dynamics (CG-MD) simulations were performed using the MARTINI forcefield as described previously [36], [56], [57]. This employs an approximately 4∶1 mapping of non-H atoms to CG particles was used. Inter-particle interactions were treated with Lennard Jones interactions between 4 classes of particles; polar (P), charged (Q), mixed polar/apolar (N) and hydrophobic apolar (C). These were then split into subtypes to reflect differing hydrogen bonding capabilities or polarity. In MARTINI P and C particle types were subdivided to reflect varying degrees of polarity. Interactions in each case were based on a lookup table, with 9 levels in MARTINI. Short range electrostatic interactions were treated Coulombically, shifted to zero between 0 and 12 Å. Lennard Jones interactions were shifted to zero between 9 and 12 Å. The integrity of the α-helices was maintained via dihedral restraints. The peptide termini were treated as uncharged.

Simulations were performed using Gromacs 3.3 (www.gromacs.org) [58]. Temperature was coupled using a Berendsen thermostat at 323 K (τ_T_ = 1 ps), and pressure was coupled semi-isotropically (across XY/Z) at 1 bar (compressibility = 3×10^−5^ bar−1, τ_P_ = 10 ps). The initial simulation timestep was 20 fs.

### Protein and lipids

Initial models of the TM α-helices were generated as ideal, atomistically detailed a-helices using standard backbone angles and side- chain conformers. These are then converted to coarse grained as described previously Around 128 DPPC molecules were used in each simulation along with around 3000 CG water particles, giving a final system size of ∼65×65×13 Å^3^.

### Mutants and phenotypes

Sequences for characterised mutations were taken from [35], [36], [37], [38] [20], [43], [44]. The first results generated experimentally for Tar were the arginine scanning mutations [45], followed by the tryptophan point mutations [43]. These were later extended to an asymmetric scan of the tryptophan/tyrosine pair at the cytoplasmic side of the membrane [44]. These were preceeded by the genetic screen of Trg which revealed a range of behaviours [20].

Single point and double interfacial tryptophan to alanine mutants (W192A, W209A and WAWA) in Tar from *E. coli* [35] reduced swarm expansion rates in aspartate, maltose and glycerol, with W209A (the cytoplasmic tryptophan) mutation with the most significant reduction in swarm expansion. W209A and WAWA mutants also were found to have reduced CheY phosphate levels, indicating a reduced CheA kinase activity, and an increased methylation state of the receptors (both in the presence and absence of aspartate and nickel).

Tryptophan/tyrosine scanning mutations of *E. coli* Tar [36] were characterised by measurement of flagellar rotation and reversal frequency, methylation states of the mutant receptors, sensitivity of the receptor to aspartate (through cell swarming behaviour and *in vitro* assays), and *in vitro* kinase activation. WY mutants show a correlation between position of the WY pair in the helix and bias in rotation direction; specifically WY mutations which reposition the pair towards the periplasm create a counter clockwise bias, and WY mutations which reposition the pair towards the cytoplasm create a clockwise bias. Furthermore, WY−3, WY+2 and WY+3 have a reduced mean reversal rate relative to the wild type, indicating that methylation may not be compensating for the degree of perturbation of the signalling state induced by the mutations. Deletion of CheR/CheB from the system (responsible for adaptive methylation) increases the flagellar rotational biasing effects of the mutants, indicating that without the methylation apparatus, the effects of the mutations could not be compensated for. WY−1, WY−2, WY−3 mutations all caused increased methylation, and WY+1, WY+2, WY+3 mutations reduced methylation. All mutants were found to be less responsive to aspartate through swarm assays, and WY+1, WY+2, and WY+3 were found to have higher Ki values *in vitro*. *In vitro* kinase assays (in the absence of CheR/CheB) showed that WY−3, WY−2, WY−1 had a strongly reduced kinase activity, and WY+2 and WY+3 unexpectedly had a slightly reduced kinase activity.

Arginine mutations of *S. typhimurium* Tar [37] were characterised in terms of kinase and methylation activity using *in vitro* assays. F189R and W192R were found to have increased kinase activity and reduced methylation activity, whilst W209R was found to have reduced kinase activity and increased methylation activity.

Mutations of *E. coli* Trg [38] were identified using a genetic screen and characterised by examination of the formation of chemotactic rings on semi solid media containing galactose, ribose or tryptone, the ability of the cells to migrate in a spatial gradient, and the methylation status of the receptor (assessed by electrophoretic mobility). L216K was found to have increased methylation (and was classed “induced”) and T215K and T215R were found to have reduced methylation (as was classed as “reduced”).

### Sidekick

The Sidekick high throughput approach is applied as described previously [25], [42]. Briefly, each helix sequence was simulated 100×100 ns. The approach described here is distinct from previous high throughput approaches [41] in several features. The simulation pipeline is entirely automated, with simulations systems built, multiple simulations performed across a cluster, and the resulting simulation trajectories analysed from an input of a sequence and key simulation variables (temperature, forcefield, simulation length etc.). Simulations were performed over a mixed computational grid consisting of a dedicated 56 core MacOS cluster and workstations. Sidekick is written in python using the numpy and matplotlib libraries for calculations and plotting graphics. Xgrid is used to distribute calculations across the grid.

## Supporting Information

Figure S1 **Tar TM2 C-terminal aromatic scanning mutants.** **A** Helix tilts with respect to the bilayer normal of the mutant TM2 helices. Kinase inactive mutants (red) mostly show an increased tilt relative to wild type, and kinase active mutants (blue) are mostly unaltered relative to wild type. **B** Helix rotations with respect to the bilayer centre of the mutant TM2 helices. Kinase inactive mutants (red), and kinase active mutants (blue) show no clear patterns.
(DOC)

Figure S2 **Position and orientation of Tar TM2 tryptophan to alanine mutations.** **A** Helix displacements with respect to the bilayer center of the mutant TM2 helices. Kinase inactive mutants (red), and mixed kinase active/inactive mutants (purple) are mostly unaltered relative to wild type. **B** Helix rotations with respect to the bilayer centre of the mutant TM2 helices. Kinase inactive mutants (red), and mixed kinase active/inactive mutants (purple) show no clear patterns.
(DOC)

Figure S3 **Tar TM2 arginine scanning mutants.** Helix rotations with respect to the bilayer centre of the mutant TM2 helices. Kinase inactive mutants (red), and kinase active mutants (blue) show no clear patterns.
(DOC)

Table S1 **Helix sequences analysed in this study.**
(DOC)
