# Supplementary figures and images for: Transmembrane Helix Dynamics of Bacterial Chemoreceptors Supports a Piston Model of Signalling
Source: PLoS Comput Biol. 2011 Oct 20;7(10):e1002204. doi: 10.1371/journal.pcbi.1002204 (PMC3197627; doi:10.1371/journal.pcbi.1002204)

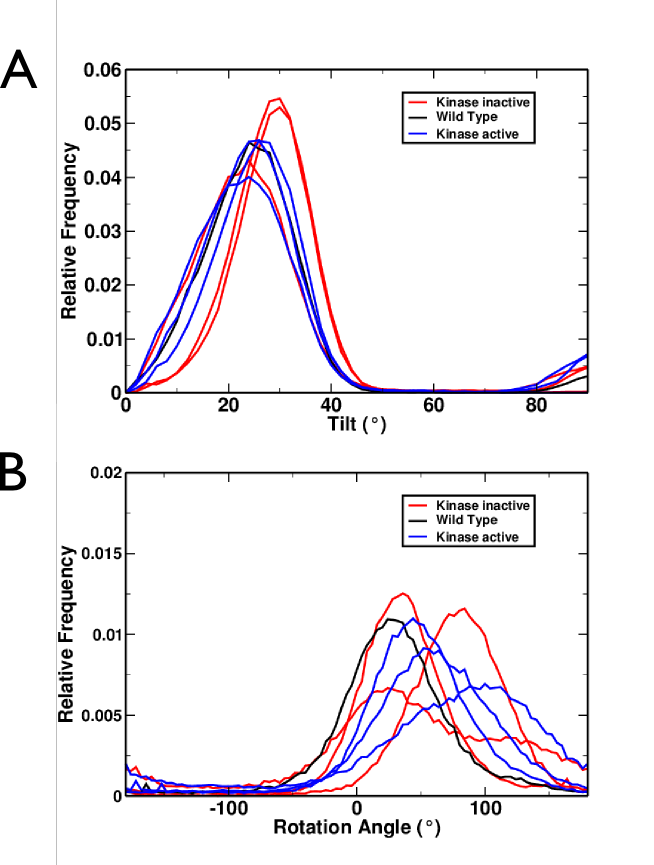


*Figure 1*

Supplement: Figure S1 — Tar TM2 C-terminal aromatic scanning mutants. A Helix tilts with respect to the bilayer normal of the mutant TM2 helices. Kinase inactive mutants (red) mostly show an increased tilt relative to wild type, and kinase active mutants (blue) are mostly unaltered relative to wild type. B Helix rotations with respect to the bilayer centre of the mutant TM2 helices. Kinase inactive mutants (red), and kinase active mutants (blue) show no clear patterns. (DOC) [file pcbi.1002204.s001.doc]

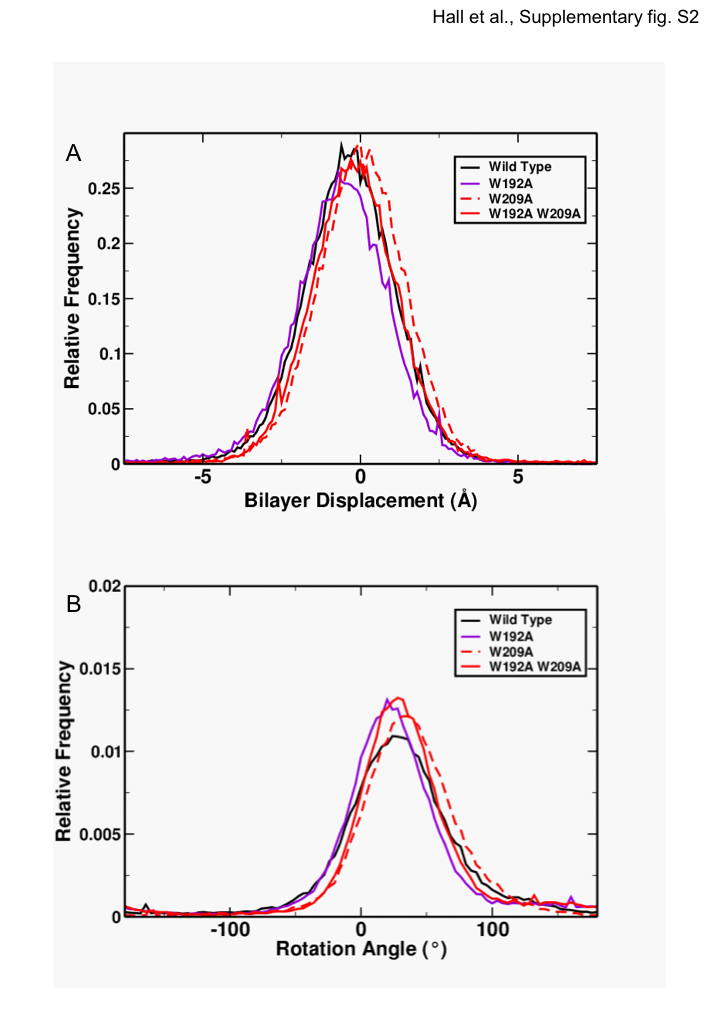


*Figure 2*

Supplement: Figure S2 — Position and orientation of Tar TM2 tryptophan to alanine mutations. A Helix displacements with respect to the bilayer center of the mutant TM2 helices. Kinase inactive mutants (red), and mixed kinase active/inactive mutants (purple) are mostly unaltered relative to wild type. B Helix rotations with respect to the bilayer centre of the mutant TM2 helices. Kinase inactive mutants (red), and mixed kinase active/inactive mutants (purple) show no clear patterns. (DOC) [file pcbi.1002204.s002.doc]

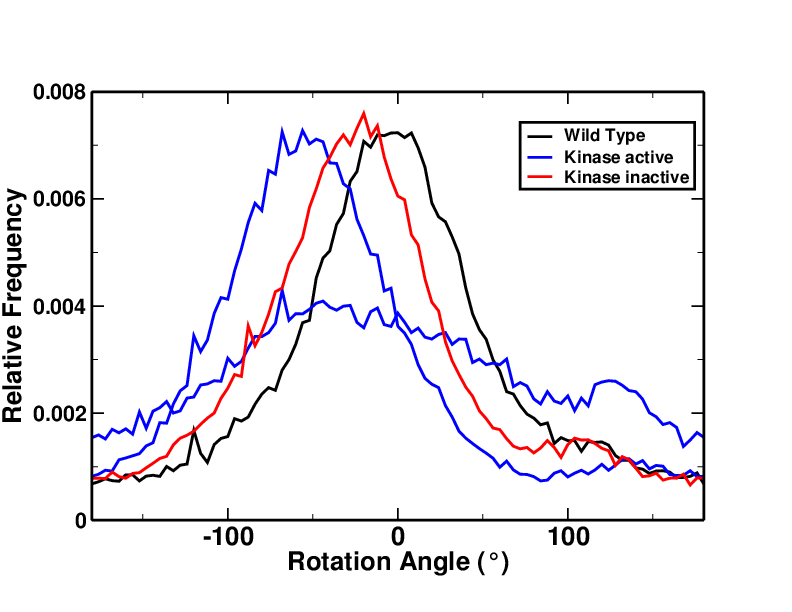


*Figure 3*

Supplement: Figure S3 — Tar TM2 arginine scanning mutants. Helix rotations with respect to the bilayer centre of the mutant TM2 helices. Kinase inactive mutants (red), and kinase active mutants (blue) show no clear patterns. (DOC) [file pcbi.1002204.s003.doc]
