# Supplementary material for: Transmembrane Helix Dynamics of Bacterial Chemoreceptors Supports a Piston Model of Signalling
Source: PLoS Comput Biol. 2011 Oct 20;7(10):e1002204. doi: 10.1371/journal.pcbi.1002204 (PMC3197627; doi:10.1371/journal.pcbi.1002204)
Supplement: Table S1 — Helix sequences analysed in this study. (DOC) [file pcbi.1002204.s004.doc]

*Supplementary Table S1: Helix sequences analysed in this study*

| **Receptor** | | **Species** | **Name** | **Sequence** | **Reference** |  |  |  |
| --- | --- | --- | --- | --- | --- | --- | --- | --- |
| Tar | *E. coli* | | Wild type | RFAQWQLAVIALVVVLILLVAWYGIRRM | 44 |  |  |  |
| Tar | *E. coli* | | WY-3 | RFAQWQLAVIALVVVLILWYLVAGIRRM | 44 |  |  |  |
| Tar | *E. coli* | | WY-2 | RFAQWQLAVIALVVVLILLWYVAGIRRM | 44 |  |  |  |
| Tar | *E. coli* | | WY-1 | RFAQWQLAVIALVVVLILLVWYAGIRRM | 44 |  |  |  |
| Tar | *E. coli* | | WY+1 | RFAQWQLAVIALVVVLILLVALWYIRRM | 44 |  |  |  |
| Tar | *E. coli* | | WY+2 | RFAQWQLAVIALVVVLILLVALVWYRRM | 44 |  |  |  |
| Tar | *E. coli* | | WY+3 | RFAQWQLAVIALVVVLILLVALVAWYRM | 44 |  |  |  |
| Tar | *E. coli* | | W192A/W209A | RFAQAQLAVIALVVVLILLVAAYGIRRM | 43 |  |  |  |
| Tar | *E. coli* | | W192A | RFAQAQLAVIALVVVLILLVAWYGIRRM | 43 |  |  |  |
| Tar | *E. coli* | | W209A | RFAQWQLAVIALVVVLILLVAAYGIRRM | 43 |  |  |  |
| Tar | *S. typhimurium* | | Wild type | RFAQWQLGVLAVVLVLILMVVWFGIRH | 45 |  |  |  |
| Tar | *S. typhimurium* | | F189R | RRAQWQLGVLAVVLVLILMVVWFGIRH | 45 |  |  |  |
| Tar | *S. typhimurium* | | W192R | RFAQRQLGVLAVVLVLILMVVWFGIRH | 45 |  |  |  |
| Tar | *S. typhimurium* | | W209R | RFAQWQLGVLAVVLVLILMVVRFGIRH | 45 |  |  |  |
| Trg | *E. coli* | | Wild type | RLGGMFMIGAFVLALVMTLITFMVLRR | 20 |  |  |  |
| Trg | *E. coli* | | L216R | RLGGMFMIGAFVLALVMTRITFMVLRR | 20 |  |  |  |
| Trg | *E. coli* | | T215R | RLGGMFMIGAFVLALVMRLITFMVLRR | 20 |  |  |  |
| Trg | *E. coli* | | T215K | RLGGMFMIGAFVLALVMKLITFMVLRR | 20 |  |  |  |
